# Management of Anticoagulant-Related Nephropathy: A Single Center Experience

**DOI:** 10.3390/jcm10040796

**Published:** 2021-02-16

**Authors:** Tanja Belčič Mikič, Nika Kojc, Maja Frelih, Andreja Aleš-Rigler, Željka Večerić-Haler

**Affiliations:** 1 Department of Nephrology, University Medical Centre Ljubljana, 1000 Ljubljana, Slovenia; tbelcic@gmail.com (T.B.M.); andreja.ales@kclj.si (A.A.-R.); 2 Institute of Pathology, Faculty of Medicine, University of Ljubljana, 1000 Ljubljana, Slovenia; nika.kojc@mf.uni-lj.si (N.K.); maja.frelih@mf.uni-lj.si (M.F.); 3 Faculty of Medicine, University of Ljubljana, 1000 Ljubljana, Slovenia

**Keywords:** anticoagulant related nephropathy, ARN, warfarin nephropathy, anticoagulant kidney disease, glomerular hemorrhage, anticoagulants, DOACs, NOACs

## Abstract

Background: Anticoagulant-related nephropathy (ARN) is a form of acute kidney injury that mainly occurs in patients with previously unrecognized glomerular disease in addition to excessive anticoagulation. Since a renal biopsy is not performed in most cases, the diagnosis is often presumptive. Methods: Here, we present the characteristics of a national Slovenian patient cohort with histologically verified ARN, from the first case in 2014 to December 2020, and a review of the current literature (Pubmed database). Results: In Slovenia, ARN has been detected in 13 patients, seven of whom were treated with coumarins, and others with direct oral anticoagulants. In seven patients, ARN appeared after excessive anticoagulation. As many as 11 patients had underlying IgA nephropathy. Similar to the global data presented here, the pathohistological impairment associated with pre-existing glomerulopathy was mild and disproportionate to the degree of functional renal impairment. The majority of our patients with ARN experienced severe deterioration of renal function associated with histological signs of accompanying acute tubular injury, interstitial edema, and occlusive red blood cell casts. These patients were treated with corticosteroids, which (in addition to supportive treatment and discontinuation of the anticoagulant drug) led to a further improvement in renal function. Conclusions: Anticoagulant therapy combined with a pre-existing glomerular injury may lead to ARN. In addition to discontinuation of the anticoagulant and supportive care, corticosteroids, which are currently listed in only a few cases in the world literature, may have a positive influence on the course of treatment. However, the benefits of steroid treatment must be weighed against the risk of complications, especially life-threatening infections.

## 1. Introduction

Anticoagulant-related nephropathy (ARN) is a recently recognized form of acute kidney injury (AKI) that is associated with rapid deterioration of kidney function and a substantially decreased survival rate [1,2]. It was first described as a complication of warfarin treatment related to an abnormal international normalized ratio (INR) characterized by glomerular hemorrhage and renal tubular obstruction by red blood cell (RBC) casts [3]. However, an abnormally elevated INR does not always cause ARN and pre-existing renal damage is required in addition to excessive anticoagulation [4]. Because renal biopsy is not performed in most cases that are overly anticoagulated, the diagnosis is often presumptive, and the prevalence of the disease is probably greatly underestimated. 

In recent years, warfarin has been partially replaced by direct oral anticoagulants (DOACs), which inhibit thrombin or factor Xa, the key proteases in the coagulation cascade. Recent reports show that, apart from warfarin, DOACs, particularly dabigatran, 85% of which is excreted through the kidneys, are also associated with ARN [5,6,7]. We present here a case of ARN related to dabigatran and warfarin, a retrospective analysis of patients with ARN in our country, and a review of the current literature.

## 2. Presentation of the Representative Case

An 82-year-old woman with a history of arterial hypertension, insulin-dependent diabetes mellitus type 2, hypothyroidism, cognitive decline, and chronic kidney disease (CKD) with a creatinine baseline value of 124 µmol/L and microscopic hematuria was admitted to the nephrology department because of AKI. Her creatinine level increased to 373 µmol/L, and urinalysis showed gross hematuria. She had signs of normocytic anemia. Her daily medication included levothyroxine, memantine, bisoprolol, rosuvastatin, and insulin. Eighteen days previously, dabigatran had been introduced at a dose of 110 mg bd due to a new onset of atrial fibrillation and a transient ischemic attack in her medical history. Laboratory, immunology, and imaging findings at admission are presented in Table 1.

**Figure 1:**
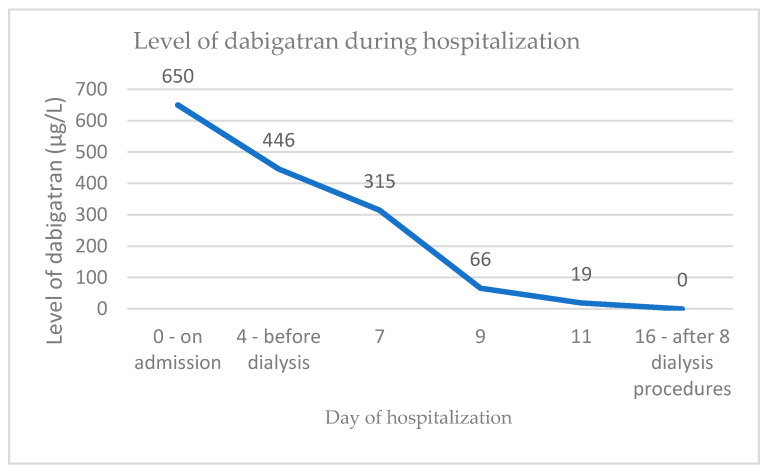
Level of dabigatran in the presented case during hospitalization.

Since gross hematuria and occult gastrointestinal bleeding were present, dabigatran was discontinued immediately. The serum concentration of dabigatran was highly elevated (650 µg/L). However, she was not treated with idarucizumab (reversal agent for dabigatran) since the bleeding was not considered life-threatening. Due to a further decrease of renal function and a high concentration of dabigatran (446 µg/L), dialysis was started. Eight dialysis procedures altogether were necessary to decrease the level of dabigatran to within the reference range (Figure 1). The thrombin time was also significantly reduced, from over 150 s, measured at admission, to 21.9 s (reference range below 21 s). 

**Figure 2:**
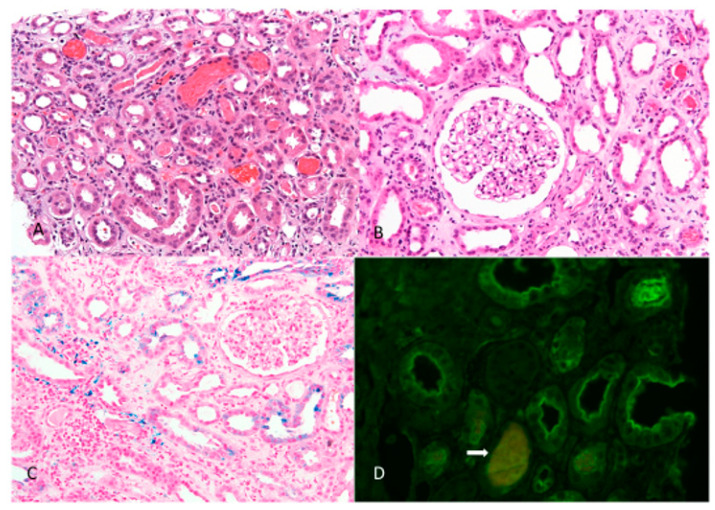
Light microscopy kidney biopsy findings: (**A**) Signs of acute tubular injury, occlusive red blood cell casts in the tubules, and diffuse interstitial edema (hematoxylin and eosin stain; original magnification × 200). (**B**) Mild glomerular segmental mesangial proliferation—mild IgA nephropathy and signs of acute tubular injury (hematoxylin and eosin stain; original magnification × 200). (**C**) Hemosiderin intracytoplasmic deposition in tubular epithelial cells (confirmed by Perl’s stain, original magnification × 200). (**D**) Occlusive red blood cell (RBC) casts not containing Tamm Horsfall protein (arrow; original magnification × 400).

Common urological causes of macroscopic hematuria were ruled out. A kidney biopsy was performed after normalization of the coagulation times. It showed signs of ARN with occlusive intratubular RBC casts, diffuse acute tubular injury, and diffuse interstitial edema, together with mild IgA nephropathy (IgAN) (Figure 2).

After the introduction of methylprednisolone (at the time-point when the patient had been dialysis-dependent for more than three weeks), kidney function gradually improved, and dialysis treatment was discontinued. Four weeks after discharge, creatinine dropped to 210 µmol/L and hematuria was markedly reduced. Methylprednisolone was gradually tapered down. Because of advanced CKD and an 11.2% risk of stroke per year (CHA2DS2-VASc score = 7), warfarin was introduced. Six months later, the patient was readmitted due to an acute decrease in kidney function and gross hematuria, this time associated with a supratherapeutic INR (4.23). Dialysis was re-started. In addition, the patient was diagnosed with a subcapsular hematoma of the left kidney (which occurred at the puncture site of a renal biopsy performed six months earlier) and required urgent embolization of a segmental artery. In the period following embolization, the patient’s clinical condition worsened, with infection of the renal subcapsular hematoma and septic shock, leading to death. An autopsy was not performed.

## 3. Patients and Methods

This presented case prompted us further to investigate cases of ARN at a national level. We therefore reviewed the medical records of our national database of kidney biopsies, consisting of 1960 native kidney biopsies over the last seven years (since our first recognized case of ARN) and updated the series with 13 cases altogether (Table 2 and Table 3). The methodology of search and analysis of selected reports in the national kidney biopsy database is presented in the Supplementary Materials.

To gain a broader insight into the global knowledge of ARN and to have the opportunity to compare the characteristics of our cohort with others, we also reviewed the global literature reported from the first mention of the disease in 2009 until 2019, including individual case reports, case series, and cohort studies. Electronic searches were conducted in the PubMed database using key words and their combinations: “anticoagulant, nephropathy, warfarin, DOAC, NOAC, CKD” (Supplementary Tables S2 and S3). 

## 4. Results

### 4.1. Clinical Features

The 13 patients (eight men and five women) with ARN in our national cohort averaged 70 years of age and had presented with gross hematuria (*n* = 11) or microscopic hematuria (*n* = 2) and acute decline of kidney function (*n* = 13) at various time-periods after the initiation of anticoagulant therapy (Table 2). A greater acute increase in serum creatinine of more than 25% from baseline was seen in 9 of 13 patients (69.2%). The anticoagulant was warfarin or acenocoumarol in seven cases, dabigatran and warfarin in one case (the presented case), dabigatran in two cases, rivaroxaban in two cases, and low molecular weight heparin (LMWH) in one case. The anticoagulant therapy was excessive in seven patients (according to INR, the concentration of dabigatran or anti-Xa-rivaroxaban). In two patients, ARN occurred late after the introduction of warfarin (10 and 15 years, respectively), in both cases associated with severe over-anticoagulation. In six patients, the indication for anticoagulation was atrial fibrillation, in four patients, a mechanical cardiac valve, and in three cases, other causes. All patients had comorbidities, including an underlying kidney disease, which was advanced glomerulosclerosis in one case, latent IgA deposits and a thin glomerular basement membrane in one case, and IgAN in 11 cases, as confirmed by the kidney biopsy. 

### 4.2. Histopathological Features

Renal biopsies from all patients with ARN showed acute tubular injury of varying intensity, including severe, moderate/severe, moderate, mild/moderate, or mild acute tubular injury (one, one, three, six, and two patients, respectively; Table 3). Eleven patients showed diffuse tubular injury, whereas only focal mild tubular injury was found in two patients. All patients presented with occlusive intratubular RBC casts without the Tam Horsfall protein in an average of 7.1% of the tubules (range 2–20.3%), which were mainly found in the distal tubules and therefore appeared to be more common in the medulla than in the cortex. Medulla was collected in 11 of 13 patients. There was no regurgitation of the Tam Horsfall protein in the urinary space of the glomeruli in any patient. In 11 of 13 cases, a diagnosis of IgAN was made, and in one case (no. 6), we observed a thin glomerular basement membrane and mesangial IgA deposition without C3 deposits. It is known that up to 16% of the healthy population have latent mesangial IgA deposits without clinical manifestations [8]. According to the Oxford classification, in all diagnosed cases of IgAN, the disease was mild with only focal mild segmental mesangial proliferation and no endocapillary or extracapillary proliferation (M0, E0, S0, C0, whereas T was not evaluated due to diffuse acute tubular injury unrelated to IgAN). In only one patient (no. 8, Table 2), there was one small fibrocellular crescent in 1 of 17 glomeruli, consistent with the Oxford classification M0, E0, S0, C1. In addition, the disease course before the episode of ARN was generally subclinical. All patients with ARN showed extensive changes in the tubulointerstitial compartment (tubular injury and occlusive intratubular RBC casts), unrelated to relatively mild active glomerular changes or glomerulosclerosis.

### 4.3. Treatment and Outcome

Details of the clinical course and treatment are presented in Table 2.

In six cases treated with anticoagulants for atrial fibrillation (patients no. 2, 3, 6, 7, 8, 9) and in two cases of post thrombotic events (patients no. 12, 13), the precipitating anticoagulant was discontinued immediately after admission. In four patients treated with warfarin or acenocoumarol for a mechanical heart valve, the excessive anticoagulant effect was reversed with vitamin K, and there was a temporary switch to heparin. Other anticoagulation reversal agents (i.e., idarucizumab for dabigatran or recombinant coagulation factor Xa for apixaban and rivaroxaban) were not used. None of our patients with ARN were treated with N-acetyl-cysteine.

Kidney biopsy was performed at a median time of 13 days after admission (range of 1 to 39 days), when safe conditions for the invasive procedure were present. Adequate reversal of anticoagulation was achieved in 14.5 ± 14.3 days (median 6.5 days) after admission in patients treated with coumarins, and in 17 ± 5.8 days (median 19 days) in patients treated with DOACs.

After histologic evidence of ARN (on average, 2 to 3 days after kidney biopsy), methylprednisolone was introduced in eight patients, with an initial dose of 0.4–0.8 mg/kg body weight (some patients initially received pulses of steroids), which was tapered over 6 to 8 weeks. 

All patients treated with steroids initially had more severe acute kidney injury, with an average increase in serum creatinine of 239.5% (range 46.5 to 489.3%) compared to an average increase of 15.82% (range 12.5 to 20.5%) in patients who were clinically judged to have no need for steroid therapy (please note that in patients no. 5 and 11, with a severe form of ARN, corticosteroid treatment was considered, but they were not treated due to the drawbacks listed below). All but one patient (no. 6) treated with steroids presented with macrohematuria. Retrospective re-evaluation of the kidney biopsy specimens revealed that the steroid-treated patients had a histologic picture of more severe acute kidney injury, including 70–100% of tubules with evidence of acute tubular injury, 40–100% interstitial edema, and an average of 9.4% of tubules filled with RBC casts. Interstitial infiltrate was rare and present in only up to 10% of the sample. In patients treated with steroids, renal function improved markedly at the time of the last follow-up compared to the time of diagnosis. 

Five patients (patients no. 3, 5, 10, 11, 13) were not treated with steroids. According to our clinical judgement, three of them lacked indication since complete restitution of mild renal dysfunction and regression of hematuria was achieved after discontinuation of anticoagulant therapy. One patient was not further treated because of already advanced glomerulosclerosis with no prospect of improvement (patient no. 5) and one had contraindications for steroids associated with comorbidities, such as an aortic stent graft infection (patient no. 11). In the three patients who presented with mild AKI and hematuria (one had microscopic hematuria and two had macrohematuria), histologic reevaluation showed a mild degree of acute tubular injury with 20–50% injured tubules, with up to 10% interstitial infiltrate and 5–25% interstitial edema. RBC casts were detected in an average of 3.9% of tubules.

A total of two patients required supportive therapy with dialysis during follow-up, including patient no. 9 (case presented), whose kidney function improved after therapy with methylprednisolone, and patient no. 5, who developed end-stage renal failure and required maintenance dialysis. One patient died due to infectious complications (case presented). No serious adverse effects were reported in the other steroid-treated patients. 

**Figure 3:**
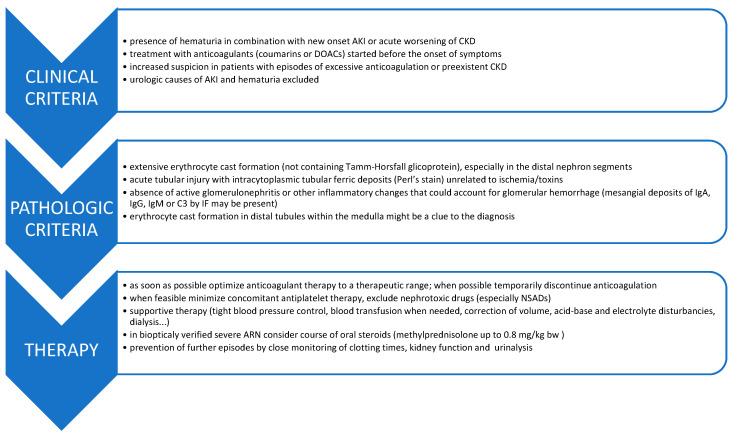
Proposed clinico-pathologic flow-chart to approach anticoagulant-related nephropathy. Abbreviations: ARN-anticoagulant related nephropathy, CKD-chronic kidney disease, AKI-acute kidney injury, DOACs-direct oral anticoagulants, IF-immunofluorescence, Ig-immunoglobulin, C3-complement component C3.

Bringing together our clinical and pathological findings with the previously reported experiences of other groups [9,10,11], we propose a useful clinicopathological algorithm to address ARN (Figure 3).

## 5. Discussion

Almost half a century has passed since an Australian scientific group led by Dr. Kincaid-Smith attempted to treat IgAN with the famous triple therapy known as the Melbourne Cocktail [12]. The triple therapy, which consisted of cyclophosphamide, antiplatelet dipyridamole and warfarin in full anticoagulant doses, would probably be considered controversial today. It proved effective in reducing proteinuria, but patients experienced worsening of renal function with more glomerular bleeding, as evidenced by increased urinary RBC counts compared to the control group [13]. 

Although this old study did not provide a revolutionary solution for our most common nephropathy, it inadvertently led to hemorrhagic adverse effects, which were not recognized until much later as being associated with warfarin (and possibly also dipyridamole). Namely, in 2009, Brodsky et al. [3] were the first to present a case series of nine patients with unexplained AKI and hematuria while on warfarin therapy. All patients included in this retrospective study had underlying kidney disease, the most common being IgAN, followed by diabetic nephropathy, lupus glomerulonephritis, focal segmental glomerulosclerosis, and nephrosclerosis [3]. Since the publication of this case series, unexplained AKI, together with excessive anticoagulation and specific renal biopsy findings with glomerular hemorrhage and occlusive RBC casts, has been defined as warfarin-related nephropathy [2]. In recent years, it has been recognized that DOACs, and even antiplatelets can also cause this type of kidney injury, and the term anticoagulant-related nephropathy (ARN) is now widely used.

By way of introduction, we present an illustrative case of a patient with recurrent ARN that initially occurred after anticoagulation with dabigatran and later recurred after DOAC was replaced with warfarin. In both cases, ARN was related to excessive anticoagulation. The patient had preexisting IgAN presenting as microscopic hematuria and renal insufficiency with an eGFR of 35 mL/min/1.73 m^2^, which seriously approached the limit at which dabigatran is contraindicated (i.e., an eGFR < 30 mL/min/1.73 m^2^). Histologic examination revealed diffuse acute tubular injury along with occlusive tubular RBC casts, consistent with ARN (Figure 2). Although dabigatran was immediately discontinued, its anticoagulant effects persisted due to severe accumulation, which maintained macrohematuria and delayed the time to kidney biopsy. The patient successfully overcame the period of renal failure with hemodialysis, which also reduced (by increasing drug excretion) the risk of life-threatening bleeding. Steroid treatment, initiated after the patient had been dialysis-dependent for more than three weeks without recovery of renal function, was considered successful in providing rapid improvement in renal function. However, the patient subsequently succumbed to sepsis, and immunosuppressive therapy may have played a role in this.

Since there are still no guidelines for the management of ARN, and in order to assess this area comprehensively, we analyzed our national cases of ARN since our first case in 2014 (Table 2 and Table 3) and reviewed the international literature reported to date (Supplementary Table S2 and Table S3). Based on our experience and including suggestions previously put forward by others [9,10,11], we propose an algorithm of diagnostic–therapeutic steps required in patients with suspected ARN (Figure 3).

In accordance with the characteristics of our national patient cohort, the most common underlying kidney disease in patients with ARN at the global level was IgAN. Other diseases, such as postinfectious glomerulonephritis, diabetic nephropathy, hypertensive nephroangiosclerosis, chronic interstitial nephritis, and vasculitis (ANCA-associated and Behcet’s disease) have rarely been reported. ARN developed in patients taking warfarin or DOACs, but was also documented in one case of dual antiplatelet therapy [14]. Interestingly, almost half of all ARN cases published in recent years were related to DOACs, especially dabigatran. These data are consistent with the results of recent analyses extracted from the International Pharmacovigilance Registry, which show that the reported annual rate of renal adverse events is almost 10 times higher for DOACs (7725 cases in 15 years) than for antivitamin K drugs (2145 cases reported in 50 years). According to this database, dabigatran and rivaroxaban are the drugs associated with a higher proportion of kidney-related adverse events, being 4.6% and 3.5%, respectively [15]. In the set of clinical cases of ARN, the median age of patients treated with dabigatran and rivaroxaban was 78 years. Apart from older age, most of the patients had more preexisting risk factors previously associated with ARN, the most common being diabetes, hypertension, heart failure, and CKD [1,2,3,16,17]. Only two patients had presumed normal kidney function before the anticoagulant was introduced, while most patients had CKD stage 2 or 3. The lowest-reported eGFR in one patient at the start of dabigatran therapy was the same as in the patient presented in our case, that is, 35 mL/min/1.73 m^2^ [18].

The risk of developing the disease is undoubtedly much higher in over-anticoagulated patients, but ARN is not always related to excessive anticoagulation [1]. According to the data of our national cohort, two patients with histologically proven warfarin associated ARN had an INR that was even below the therapeutic range at the time of ARN diagnosis. Based on these observations and animal studies of ARN [19], it has been suggested that excessive anticoagulation alone is not sufficient. It is consequently now believed that for significant renal bleeding to develop, the glomeruli must be vulnerable to glomerular hemorrhage.

Based on the national and international data we have collected, the most common susceptibility to the disease appears to be due to a preexisting glomerulopathy, particularly IgAN. It appears that IgAN is exceptionally susceptible to anticoagulant-induced glomerular hemorrhage, but the reason for this remains poorly understood. In patients with IgAN, glomerular changes are characterized by hematuria caused by leakage of erythrocytes through the glomerular basement membrane into the urinary space, resulting in the formation of occlusive RBC casts in the tubules. As confirmed by our study, in contrast to the histological presentation of mild IgAN, much more numerous RBC casts are seen in patients with concomitant ARN. Namely, of 347 preimplantation kidney biopsies performed at our transplant centre over the past six years, we found IgA deposits consistent with IgAN (staining for IgA at IF ≥1+) in 50 (14.4%) preimplantation biopsies. These patients had mild IgAN, with only focal mesangial proliferation with an Oxford classification score comparable to the group of patients with ARN and underlying IgAN presented here (unpublished data). However, scarce RBC casts were detected in only 2 of 50 preimplantation biopsies with IgAN, whereas numerous RBC casts were present in all cases of ARN with underlying IgAN. Based on these data and supported by animal studies [19], we assume that more severe glomerular hemorrhage seen in patients with ARN, with extensive tubular obstruction by RBC casts, is not due to mild glomerular injury, but rather provoked by (usually) overt anticoagulation. After glomerular hemorrhage, further exacerbation of renal injury is associated with direct damage to tubular epithelial cells by oxidative stress, in which free hemoglobin binds to receptors in tubular cells and activates the formation of reactive oxygen species and an inflammatory response (16). The latter is consistent with the detailed findings of the pathohistological analysis of our patients, in whom the degree of renal injury appears to depend more on the extent of tubular damage, the number of occlusive RBC casts and accompanying interstitial edema than on the severity of the underlying glomerulopathy. 

To detect ARN in patients receiving anticoagulants, we examined kidney biopsies from all patients treated with anticoagulant therapy or receiving anticoagulant prophylaxis before biopsy (Supplementary Materials). Histological reexamination revealed various renal diseases, but in none of these cases did we find a specific pattern of acute tubular injury and occlusive RBC casts characteristic of ARN, which could not be explained by concomitant glomerulonephritis. However, in biopsies with extensive glomerular changes, including endocapillary proliferation and crescents in ANCA vasculitis or immune complex glomerulonephritis, possible incidental signs of ARN may go undetected due to glomerulonephritis-related RBC casts and tubular injury, and these patients require thorough monitoring of kidney disease and anticoagulant therapy. According to the results of our study, ARN is histologically characterized by a specific pattern of acute tubular injury and occlusive RBC tubular casts combined with mild glomerular changes that are disproportionate to the glomerular injury. Indeed, other entities, including mild IgAN on preimplantation biopsies or kidney biopsy findings from patients on anticoagulant therapy, do not fulfill both features of kidney damage. However, it is important to emphasize that in the case of a suboptimal renal biopsy without medulla, where the majority of RBC casts were usually detected, only acute tubular injury may be detected and ARN overlooked. Although an experienced pathologist might suggest ARN, an accurate diagnosis still requires a thorough clinicopathologic correlation focusing on the patient’s medication, gross hematuria, and clinical course of kidney disease. Thus, a representative kidney biopsy specimen and accurate clinical data are therefore both indispensable factors to correctly diagnose ARN.

After supportive measures and reversal of anticoagulation, renal function and hematuria were restored in three patients (patients no. 3, 10, 13), with ARN presenting as mild renal insufficiency and associated histology showing mild acute tubular injury and interstitial edema. Supportive measures with temporary complete discontinuation of anticoagulant therapy, when possible, or at least strict optimization of anticoagulant therapy, is thus probably a sufficient step to enable a favorable outcome in mild clinico-pathologic forms of ARN. One patient with advanced glomerulosclerosis (with no prospect of improvement) was not further treated. This was the only patient who progressed to end-stage renal failure. One patient with a severe form of ARN and multiple comorbidities (patient no. 11) could not be treated with steroids due to contra-indications. His renal function improved only slightly after conservative measures (eGFR increased from 7 to 15 mL/min/1.73 m^2^). This patient left the hospital of his own free will, and was lost for follow-up. Importantly, anticoagulant therapy could not be discontinued in either of these two cases because of a mechanical aortic valve.

Among our ARN patients, 8 of 13 (61.5%) were treated with steroids. In the steroid-treated patients, renal function had improved markedly at the time of the last follow-up visit compared with the time of diagnosis. Because methylprednisolone was not introduced until after the renal histology report was obtained, the median time from admission to treatment was longer in patients treated with DOACs than in patients treated with coumarins (19 vs. 6.5 days), in whom more time was needed to meet the hemostatic criteria for performing a safe biopsy. In an overall view of the data obtained, the improvement in renal function seems to be slightly better in ARN patients treated with coumarins than those receiving DOACs (also taking into account the duration of follow-up). However, either a direct relationship between the duration of excessive anticoagulation and the severity and persistence of tubular injury, or a possible association of a more favorable outcome in the case of earlier introduction of methylprednisolone, was difficult to establish in our small, heterogenous group of patients.

Although early administration of steroids has been reported to accelerate recovery from the more classic inflammation observed in drug (anticoagulants)-induced nephritis [20,21,22,23,24], the use of corticosteroids in ARN is still limited to a few reported cases [25,26]. Our decision to treat with steroids was based on clinical judgement and the extent of pathohistological changes associated with acute tubular injury. Indeed, subsequent retrospective insight shows that, in our case series, steroids were used to treat those ARN patients who had a clinically severe, non-resolving form of acute kidney injury, reflected by an average 239% increase in serum creatinine and usually presenting with macrohematuria. Consistent with the clinical picture, these patients had histological signs of diffuse moderate or moderate/severe acute tubular injury in 70–100% of tubules, with extensive (40–100%) interstitial edema and a markedly higher number of obstructive RBC casts. At the same time, the glomerular tufts were without appreciable proliferative lesions.

Inevitably, we find it necessary to mention that in the current absence of clear recommendations for the treatment of ARN, the decision to introduce steroids into the therapeutic protocol was largely based on the positive historical experience of our center in IgAN patients with AKI and macroscopic hematuria. Indeed, in 2009, Kveder et al. [27] observed signs of acute tubular necrosis and foci of interstitial nephritis (especially near the areas with the most extensive erythrocyte or hemoglobin casts) in a group of patients with IgAN and associated macromaturia. Regardless of the pathogenesis of such findings in patients with IgAN (please note that at that time, we were not familiar with ARN and anticoagulant therapy was not addressed), it was at this time that we gained our first insights into the possible tubulotoxic and inflammatory effects of occlusive erythrocyte and hemoglobin casts and realized possible favorable outcomes following corticosteroid treatment. In subsequent years, the etiopathogenesis of RBC cast-associated tubular injury has been attributed to the deleterious local effects of catalytic iron released from decaying erythrocytes. The latter is thought to stimulate excessive production of hydroxyl radicals, leading to damage to the lipoprotein components of tubular cell membranes and ultimately to apoptosis/necrosis of the tubular cells [9,10]. Similar oxidative stress-mediated damage may also occur in some other conditions, such as ischemia-reperfusion injury [28,29] or cholesterol atheroembolism [30], in which corticosteroids have been shown to ameliorate the associated tubulointerstitial injury and prevent the progression to irreversible fibrosis. Nevertheless, the results of our ARN patients are favorable, since only one patient required maintenance dialysis (7.7%), whereas in a previous series of histologically verified ARN, a much larger proportion of patients with ARN (66.7%) did not recover renal function [3].

At this point, however, it is important to recall the introductory case of a patient with ARN in whom renal function improved after the introduction of a steroid, but the outcome was fatal due to subsequent infection. In order to gain full insight into the benefits and risks of steroids in the ARN population, a prospective controlled study is therefore needed.

## 6. Conclusions

Anticoagulant therapy together with pre-existing glomerular injury may lead to ARN. This may occur regardless of the type of anticoagulant used, and is not necessarily associated with excessive anticoagulation. ARN is nowadays more often associated with DOACs, especially dabigatran. 

For gross hematuria with worsening kidney function in a patient on anticoagulant therapy, ARN is strongly suggested and should be managed promptly. 

Treatment of ARN is mainly supportive and includes normalization of clotting times to a therapeutic range. The role of steroids is still debatable, but they have successfully been used for management in the majority of our reported cases. However, the benefits of steroid treatment must be weighed against the risk of complications, especially life-threatening infections. 

Currently, the most reasonable therapeutic approach in ARN is prevention. In view of the increased risks in patients with CKD, especially IgAN, kidney function and urinalysis should be performed in all patients before the initiation of anticoagulant therapy, and closely monitored thereafter. 

## Figures and Tables

**Table 1:** Laboratory and ultrasonography findings in the presented patient on admission.

Laboratory Analysis	
Sodium	119 mmol/L
Potassium	5.2 mmol/L
Urea	15.2 mmol/L
Creatinine	373 µmol/L
eGFR (CKD-EPI)	10 mL/min/1.73m^2^
pH	7.33
Sodium bicarbonate	18.3 mmol/L
Hemoglobin	78 g/L
Mean corpuscular volume	88.5
aPTT	141 s
TT	>150 s
TT-dabigatran	650 µg/L
**Urine analysis**	
**Urine sediment**	
Leukocytes	>100 per HPF
Bacteria	0
Red blood cells	>100 per HPF
Casts	Rare per HPF
**Immunologic findings**	
ANCA, anti-GBM Ab, ANA	All negative
**Doppler ultrasound**	
Imaging result	Normal-sized kidney with preserved parenchymal thickness, increased echogenicity and increased RI, no signs of obstruction

Abbreviations: eGRF (CKD-EPI), estimated glomerular filtration rate using the Chronic Kidney Disease Epidemiology Collaboration equation; aPTT, activated partial thromboplastin time; INR, international normalized ratio; TT, thrombin time; TT-dabigatran, the level of dabigatran; HPF, high power field; ANCA, anti-neutrophil cytoplasmic antibody; anti-GBM Ab, anti-glomerular basement membrane antibody; ANA, anti-nuclear antibody; RI, resistance index.

**Table 2:** Clinical and demographic data in a Slovenian cohort of patients with anticoagulant-related nephropathy.

Pt	Age	Sex	AC	SCr Baseline (µmol/L)	SCr at Biopsy (µmol/L)/eGF (mL/min)	Increase in Serum SCr from Basal Level (%)	SCr at Discharge (µmol/L)/eGF (mL/min)	SCr (µmol/L)/eGF (mL/min) at Time of Last Follow up/Follow up Time	Time to ARN from Introduction of AC	Symptoms at Biopsy	INR/aPTT (s)/Dabigatran level (µg/L)/Anti-Xa-Rivaroxaban (µg/L)	Relevant Comorbidities	Treatment	Outcome
1	77	f	warfarin	not known	448 (10)	>25	285 (13)	127 (35)/17 months	20 months	gross hematuria	supra-therapeutic INR was never recorded	mitral and aortic valve stenosis, post CVI, meningeoma, recent breast cancer	discontinuation of warfarin, methyl-prednisolone 3 × 125 mg iv, followed by 0.5 mg/kg bw for 1 month, then taper down until discontinuation in six weeks	improvement of kidney function, no rea-appearance of gross hematuria, later aceno-coumarol was introduced with no ARN
2	74	m	warfarin	150	510 (9)	240	337 (16)	137 (43)/21 months	2 years	gross hematuria	INR 3.8	atrial fibrillation, aortic stenosis, congestive heart failure, Parkinson’s disease	discontinuation of warfarin; methyl-prednisolone 0.5 mg/kg bw for 1 month, then taper down until discontinuation in 6 months	improvement of kidney function, no hematuria
3	66	f	warfarin	110	first episode 129 (37), second episode 160 (28)	first episode 17, second episode 45.4	120 (39)	126 (37)/43 months	3 months	gross hematuria	INR 1.63	atrial fibrillation, Sjogren’s syndrome, fibromyalgia	discontinuation of warfarin; on re-introducement gross hematuria re-appeared with an INR 3.6, after that event warfarin was stopped	improvement of kidney function, persistent micro-hematuria
4	51	m	warfarin	170–200	249 (24)	46.5	253 (23)	174 (36)/15 months	15 years	gross hematuria	INR 5.0	mitral valve replacement, atrial fibrillation, CVI, diabetes mellitus type 2, cystostoma due to urethral stenosis	temporary discontinuation of warfarin, LMWH introduced; steroids 0.7 mg/kg bw 1 month, then taper down until discontinuation in 3 months; strict control of anticoagulant therapy	improvement of kidney function, hematuria persisted; recurrence of gross hematuria after reintroduction of warfarin (at INR 5.0), improvement after correction of INR
5	76	m	warfarin	430	487 (9)	13.30	on dialysis	on dialysis/12 months	10 years	gross hematuria	INR 4.4	mechanical aortic valve, severe atherosclerosis, arterial hypertension, diabetes mellitus type 2, HBV infection	dialysis, better control of INR	no improvement of kidney function
6	69	m	dabigatran, 2 × 150 mg	88	194 (30)	120.5	181 (32)	164 (36)/6 months	2 months	microscopic hematuria	dabigatran level 293 µg/L	atrial fibrillation, arterial hypertension, diabetes mellitus type 2, ethylic liver cirrhosis	discontinuation of dabigatran, methyl-prednisolone, 0.6 mg/kg bw 14 days, then taper down until discontinuation in 3 months	improvement of kidney function, persistent microhematuria
7	67	m	rivaroxaban (previously on apixaban and dabigatran which caused GI bleeding)	75	442 (12)	489.3	386 (13)	82 (86)/12 months	2.5 months	gross hematuria	INR 1.24, aPTT 45.1 s	atrial fibrillation, IgA vasculitis with skin involvement	discontinuation of rivaroxaban, methyl-prednisolone, 3 × 250 mg iv, followed by 0.8 mg/kg bw 1 month, then taper down until discontinuation in 4 months	improvement of kidney function
8	82	f	rivaroxaban (previously on apixaban for 5 days which was stopped due to skin rash)	119	315 (12)	164.7	267 (15)	179 (22)/15 months	1 week	gross hematuria	INR 1.65, aPTT 41.4 s, antiXa- rivaroxaban 28 µg/L -performed 21 days after rivaroxaban discontinuation	atrial fibrillation, arterial hypertension, ischemic heart disease	discontinuation of rivaroxaban, methyl-prednisolone, three pulses 500 mg, followed by 0.7 mg/kg bw 1 month, then taper down until discontinuation in 3 months	improvement of kidney function, no hematuria
9	82	f	dabigatran, 2 × 110 mg	124	373 (10)	200.8	401 (9)	transient improvement of SCr 201 µmol/L, however numerous further complications (please see case report for details)	18 days	gross hematuria	650 µg/L	atrial fibrillation, diabetes mellitus type 2, arterial hypertension	discontinuation of dabigatran, methylprednisolone.4 mg/kg bw 1 month, then taper down until discontinuation in 3 months	improvement of kidney function, later re-bleeding on warfarin, death due to sepsis
10	56	f	acenocoumarol (skin rash on warfarin)	72 (82)	81 (70)	12.5	95 (53)	80 (71)	11 months	microscopic hematuria	2.42	mechanical aortic valve, atrial fibrillation, systemic lupus, Sjogren’s syndrome, IgA vasculitis with isolated skin involvement, kidney stone	better control of INR in the lower level of therapeutic range	persistence of microscopic hematuria, stable kidney function
11	66	m	gross hematuria appeared after 8 days of LMWH therapeuric dose -previously aceno-coumarol	149 (42)	669 (7)	349	350 (15)	unknown, the patient refused further treatment	26 years aceno-coumarol, LMWH 8 days	gross hematuria, epistaxis	INR 1.36, anti-Xa LMWH 0.69	mechanical aortic valve, IgA vasculitis with predominant skin involvement, SCr 108 µmol/L and micro-hematuria treated with steroid prior to current hospitalization, infection of aortic stent graft, post CVI	patient transiently converted to heparin; due to aortic stent graft infection steroid treatment was contraindicated	gross hematuria resolved after LMWH was discontinued and patient was converted to heparin, kidney function slightly improved, acenocoumarol was reintroduced with no additionalbleeding episodes
12	60	m	warfarin	74	372 (14)	402.7	274 (21)	206 (29)/1 month	3 months	gross hematuria	INR 3.46	arterial hypertension, portal vein thrombosis	methyl-prednisolone i.v. pulses 3 × 500 mg, then 0.8 mg/kg bw for 1 month (still on therapy), anticoagulant stopped	improvement of kidney function
13	81	m	warfarin 13 years converted to dabigatran 3 weeks prior to biopsy	117 (53)	141 (40)	20.5	114 (52)	117 (50)/12 months	3 weeks post introduction of dabigatran	gross hematuria	INR 1.31, aPTT 78.1 s, dabigatran level 65 µg/L	chronic heart failure, chronic Budd Chiarri, post cerebrovascular insult	discontinuation of dabigatran	improvement to baseline function in three weeks

Abbreviations: Pt, patient; AC, anticoagulant; SCr, serum creatinine; eGFR, estimated glomerular filtration rate; ARN, anticoagulant related nephropathy; INR, international normalized ratio; aPTT, activated partial thromboplastin time; CVI, cerebrovascular insult; HBV, hepatitis B virus; IgA, immunoglobulin A; LMWH, low molecular weight heparin.

**Table 3:** Histological findings in kidney biopsies in a Slovenian cohort of patients with anticoagulant-related nephropathy.

Patient No.	Kidney Biopsy	Oxford Classification	EM–GBM	Podocyte Effacement	RBC Casts	Global Glomerulo-Sclerosis	ATI	ATI Degree	Interstitial Edema	Interstitial Infiltrate	Perls	IFTA
1	ARN, IgAN	M0, E0, S1, T0, C0	185–370 nm, average 280 nm	no	5.70%	7/20	100%	moderate	100%	none	tubuli ++ int. +	15%
2	ARN, IgAN	M0, E0, S1, T0, C0	230–505 nm, average 350 nm	no	9.30%	5/23	90%	moderate/severe	90%	5%	tubuli + int. +	15%
3	ARN, IgAN	M0, E0, S1, T0, C0	230–385 nm, average 325 nm	no	6.40%	0/6	50%	mild	10%	none	tubuli ± int. −	10%
4	ARN, IgAN	M0, E0, S1, T2, C0	220–400 nm, average 300 nm	30%	7.80%	4/29	70%	mild	70%	up to 10%	tubuli ++ int. ±	20%
5	ARN, 50% global glomerular sclerosis, unclassified	/	230–515 nm, average 370 nm	no	3.20%	7/14	70%	mild/moderate	40%	none	tubuli ± int. −	20%
6	ARN, thin GBM, latent IgA deposits	M0, E0, S0, T0, C0	155–265 nm, average 190 nm	20%	7%	2/12	50%	mild	25%	up to 5%	tubuli +/++ int. −	15%
7	ARN, IgAN	M0, E0, S0, T0, C0	165–425 nm, average 285 nm	15%	10%	1/18	90%	moderate	90%	up to 10%	tubuli + int. +	up to 10%
8	ARN, IgAN	M0, E0, S0, T1, C1	210–440 nm, average 325 nm	not aplicable due to glomerular colapse	4.80%	3/17	50%	mild	20%	up to 10%	tubuli ± int. +	10%
9 -case report	ARN, IgAN	M0, E0, S0, T0, C0	165–475 nm, average 320 nm	not applicable due to glomerular collapse	8.10%	1/11	100%	severe	100%	5%	tubuli +++ int. ++	10%
10	ARN, IgAN	M0, E0, S0, T0, C0	120–305 nm, average 200 nm	20%	2%	1/10	25%	mild	25%	none	tubuli ± int. −	10%
11	ARN, IgAN, benign nephrosclerosis	M0, E0, S0, T0, C0	210–415 nm, average 290 nm	10%	20.30%	1/8	100%	mild/moderate	40%	none	tubuli ± int. −	up to 5%
12	ARN, IgAN	M0, E0, S0, T0, C0	165–335 nm, average 260 nm	15%	4.90%	3/17	80%	moderate	45%	up to 10%	tubuli + int. ±	5–10%
13	ARN, IgAN	M0, E0, S0, T0, C0	225–400 nm, average 305 nm	not applicable due to glomerular collapse	3,3%	2/22	20%	mild	5%	up to 10%	not performed	20%

Abbreviations: EM–GMB, electron microscopy-glomerular basement membrane; RBC, red blood cell; ATI, acute tubular injury; IFTA, interstitial fibrosis and tubular atrophy; IgAN, IgA nephropathy; ARN, anticoagulant related nephropathy; int., interstitium.

## Data Availability

Data is contained within the article and supplementary material.

## Supplementary Materials

The following are available online at https://www.mdpi.com/2077-0383/10/4/796/s1, Table S1: Classification of patients with histological signs of diffuse tubular injury according to the associated cause suggested by clinicopathological correlation, Table S2: International ARN cases published from 2009 to 2019 (source Pubmed), Table S3: International case series with kidney biopsy findings from 2009 to 2019 (source Pubmed).
