# Supplementary material for: Management of Anticoagulant-Related Nephropathy: A Single Center Experience
Source: J Clin Med. 2021 Feb 16;10(4):796. doi: 10.3390/jcm10040796 (PMC7920283; doi:10.3390/jcm10040796)
Supplement: Supplementary file 1 [file jcm-10-00796-s001.pdf]

Supplementary material

National renal biopsy database search methodology and clinical and histological reappraisal of selected reports.

Medical records from our national database of kidney biopsy specimens from 1/1/2014 to 1/12/2020 (Institute of Pathology, Faculty of Medicine, Ljubljana, Slovenia) were included in this analysis. Institutional software was used to extrapolate selected datasets to perform clinical and histological re-evaluations. The preserved renal biopsy specimens were fixed in formalin, embedded in paraffin, sectioned, and stained in several levels according to standard histological protocol, including hematoxylin/eosin (HE)-, Periodic acid Schiff (PAS)-, Trichrome-, Van Gieson Weigert -, and silver staining. In addition, we performed Perl's stain and immunohistochemistry for Tamm Horsfall protein (Abcam, Cambridge, United Kingdom).

First, patients with a definite histologic diagnosis of ARN were extrapolated. This search returned 12 results.

To detect possible overlooked cases, we then analyzed all patients based on a histologic diagnosis of acute tubular injury, which (along with occlusive RBC tubular casts) is the basic histomorphologic pattern of ARN. A search for records with histologic signs of >50% diffuse tubular injury yielded 249 results (13.1% of biopsy samples). These patients were then re-evaluated clinically and histologically by the authors to exclude missed ARN, grouped by probable cause, and presented in **Supplementary Table 1**. Additionally, in order to detect possible misdiagnosed cases, we evaluated clinical and histological records of all patients that had kidney biopsy performed in the same time period and, prior to kidney biopsy, were treated with anticoagulant therapy or received anticoagulant prophylaxis. We found 45 such cases. These patients were then clinically and histologically re-evaluated by the authors. Nine (20%) of them had ANCA glomerulonephritis, 9 (20%) IgA nephropathy, 5 (11%) hypertensive kidney disease, 4 (9%) membranous nephropathy, 4 (9%) minimal change disease, 3 (7%) diabetic nephropathy, 3 (7%) monoclonal immunoglobulin disease, 3 (7%) immune complex glomerulonephiritis, 2 (4%) thin basement membrane disease, 1 (2%) acute tubular injury, 1 (2%) chronic interstitial nephritis and 1 (2%) atheroembolic kidney disease.

By the two means (i.e., analysis of patients treated with anticoagulants and patients with histologically proven diffuse tubular damage), we detected 1 additional case of ARN nephropathy, totaling 13 patients.

The histological lesions of the 13 patients with ARN were then re-evaluated by 2 pathologists (N.K. and M.F.) using light microscopy and immunofluorescence and scored/graded according to the Oxford classification in case of underlying IgAN. The amount of RBC casts in the renal cortex and medulla was quantitatively assessed (% of RBC casts in cortex and medulla). Clinical data and disease progression were extrapolated from medical records archived at the University Medical Center Ljubljana and reassessed by 3 nephrologists (T.B.M., Ž.V.H. and A.A.R.).

Supplementary Table 1. Classification of patients with histological signs of diffuse tubular injury according to the associated cause suggested by clinicopathological correlation.

**PATIENTS WITH DIFFUSE ACUTE TUBULAR INJURY**	**NUMBER**	**(%)**
GLOMERULONEPHRITIS	88	35.3
ANCA	40	16
IgAN	10	4
other	38	15.3
TUBULO-INTERSTITIAL NEPHRITIS	24	9.6
Drug associated	15	6

other	9	3,6
PIGMENT INDUCED	24	9.6
ARN	11	4,4
myoglobin (rabdomyolisis)	10	4
Hemoglobin, bilirubin	3	1.2
NEPHROTIC SYNDROME - RELATED	22	8.8
membranous nephropathy	15	6
minimal change disease	5	2
other (FSGS, amyloidosis)	2	0.8
PRERENAL (including sepsis)	20	8
MALIGNANCY-INDUCED	19	7.6
light chain deposition disease	14	5.6
myeloma nephropathy	3	1.2
proximal tubulopathy	2	0.8
DRUG INDUCED	15	6
TROMBOTIC MICROANGIOPATHY	13	5.2
POSTRENAL (obstruction)	5	2
HEPATORENAL SYNDROM	4	1.6
CRYSTAL INDUCED	4	1.6
INFECTION INDUCED	2	0.8
OTHER	2	0.8
UNKNOWN	6	2.4
ALL	**249**	**100**

Supplementary Table 2: International ARN cases published from 2009 to 2019 (source Pubmed).

Reference	Age	Sex	Type of AC, dose	Indication for AC treatment	Plasma dabigatran conc. (ng/ml)	INR/aPTT (s)	SCr baseline (µmol/l)	SCr at dg (µmol/l)	Clinical presentation	Time of renal injury after AC therapy	Underlying kidney disease and other illnesses	Kidney biopsy	Treatment	Outcome
**Gois M et al., 2017. [1]**	84	m	acenocoumarol	atrial fibrillation	/	6.96	88.4	413	gross hematuria, fatigue, shortness of breath	three years	IgAN *M1E0S0T0	YES: diffuse acute tubular necrosis, occlusive RBC casts, IgAN	discontinuation of acenocoumarol, enalapril to decrease proteinuria	improvement of kidney function
**Golla A et al., 2018. [2]**	50	f	acenocoumarol	artificial mitral valve	/	4.7/49.5	79.5	415	abdominal tenderness, gross hematuria	two years	chronic rheumatic heart disease	YES: acute TIN with RBC casts	steroids, discontinuation of acenocoumarol	improvement of kidney function
**Brodsky SV et al., 2017. [3]**	82	f	apixaban, 2x 2.5 mg	atrial fibrillation	/	U	288	751	oligoanuria, microscopic hematuria	ten days	high blood pressure, coronary artery disease, congestive heart failure, CKD stage III, possible ANCA associated/ post-infectious GN	YES: occlusive RBC cats in renal tubules, mild mesangial IgA and C3 deposition	discontinuation of apixaban, steroids	no improvement of kidney function, dialysis dependent
**Escoli R et al., 2015. [4]**	69	f	dabigatran, 2x110 mg	atrial fibrillation	U	2.3/68	132	707	nausea, vomiting, oliguria	two weeks	unknown	YES: intra-tubular RBC casts, interstitial hemorrhage, IgAN	dialysis, blood transfusion, discontinuation of dabigatran	improvement of kidney function
**Kalaitzidis RG et al., 2017. [5]**	78	f	dabigatran, 2x110 mg	atrial fibrillation	U	1.9/150	88.4	601	gross hematuria	one year	U	YES: RBC casts, IgAN	discontinuation of dabigatran	improvement of kidney function

**Li X et al., 2019. [6]**	61	m	dabigatran, 2x110 mg	atrial fibrillation	U	4.09/68.3	87	418	gross hematuria	one year	none	YES: diffuse ATN with RBC casts inside the renal tubules, IgAN	discontinuation of dabigatran, dialysis, steroids	improvement of kidney function
**Ikeda M et al., 2019. [7]**	67	f	dabigatran, 2x150 mg	DVT	/	2.47/96.7	44	324	gross hematuria	five years	U	YES: tubular obstruction by RBC casts, diffuse acute tubular injury, interstitial hemorrhage, IgAN	discontinuation of dabigatran	improvement of kidney function
**Sharfuddin N et al., 2018. [8]**	81	f	dabigatran, 2x150 mg	atrial fibrillation	/	1.6/ 50	91	177	dyspnea, hypoxemia, brown-colored urine	two years	U	YES: tubular RBC casts, tubular cell epithelial injury, resolving post-infectious GN	discontinuation of dabigatran, dialysis	no improvement in kidney function, remained dialysis dependent
**Krátká K et al., 2018. [9]**	82	m	dual antiplatelet therapy (acetylsalicylic acid and clopidogrel)	coronary stenting due to unstable angina pectoris	/	1.22/-	pres. normal	522	gross hematuria	eight months	U	YES: RBC casts in renal tubules, GN with monoclonal deposits due to NH-MCL	dialysis, discontinuation of dual antiplatelet therapy	worsening of kidney function, initiation of dialysis
**Fujino Y., 2019. [10]**	75	m	rivaroxaban 10 mg	atrial fibrillation	/	/	99.8	380	gross hematuria	three years	none	YES: RBC casts in renal tubules, moderate interstitial fibrosis and tubular atrophy, mild IgA vasculitis	discontinuation of rivaroxaban	no improvement in kidney function

**Oliveira et al. 2017. [11]**	82	f	rivaroxaban 20 mg	atrial fibrillation	U	2.3/-	88.4	442	gross hematuria	two months	none	YES: occlusive RBC casts, interstitial hemorrhage, hypertensive nephron-angiosclerosis, chronic interstitial nephritis	discontinuation of rivaroxaban	worsening of kidney function, dialysis
**NG Cy et al., 2016. [12]**	56	f	warfarin	atrial fibrillation and mech. mitral valve	/	4.95/-	normal kidney function	317	routine follow-up, microscopic hematuria	not known	U	YES: RBC casts, IgAN	discontinuation of warfarin, steroids	improvement of kidney function
**Bento CP et al., 2015. [13]**	74	m	warfarin	atrial fibrillation	/	U	97	306	gross hematuria	eight months	postreptoc. GN	YES: previous post-infectious GN, diabetic nephropathy, erythrocyte cylinders in the tubules	discontinuation of warfarin	worsening of kidney function, initiation of dialysis
**Behera SK et al., 2018. [14]**	29	f	warfarin	mitral stenosis and atrial fibrillation	/	1.02/-	219	U	abdominal pain, anuria	not known	none	YES: congested glomeruli with entrapped RBCs, dilated scattered tubules showing RBCs with few eosinophilic casts, moderate tubular necrosis, interstitial edema with scattered lymphocytes and occasional eosinophils	dialysis, discontinuation of warfarin	improvement of kidney function, discontinuation of dialysis

**Santos C et al., 2013. [15]**	74	m	warfarin	atrial fibrillation	/	4.6/-	159	212	nose and gum bleeding, gross hematuria	U	U	YES: diffuse mesangial proliferation, RBCs in tubular lumina with occlusive RBC casts IF staining: predominant C3 deposits (1+) and IgG (1+) in the mesangium and capillary loops with occlusive RBC casts	dialysis, vitamin K	worsening of kidney function, initiation of dialysis
**Zerah L et al., 2015. [16]**	70	m	warfarin	atrial fibrillation	/	12.8/-	55	1084	gross hematuria, fatigue	U	none	YES: intratubular occlusive RBC casts, tubular necrosis	vitamin K, discontinuation of warfarin, dialysis	improvement of kidney function
**Larpparisuth N et al., 2015. [17]**	56	m	warfarin	artificial aortic valve (endocarditis)	/	6.08/-	123	1016	gross hematuria, anorexia, fatigue	two years	previous AKI (glomerulonephritis associated with endocarditis)	YES: intratubular RBC casts, interstitial fibrosis, moderate to severe intimal fibrosis of intralobular arteries	vitamin K, discontinuation of warfarin, dialysis	improvement of kidney function

**Remková A et al., 2010. [18]**	41	m	warfarin	recurrent DVT (Behcet's disease, antiphospholipid syndrome)	/	below 4.0/-	U	U	hematuria, acute retroperitoneal hemorrhage, renal, para- and perirenal hematomas	ten days	nephritis with increased glomerular cellularity, arteriolar hyalinization and focal interstitial round-cell infiltration - renal manifestation of Behcet's disease	YES, but only of the preserved renal parenchyma of the removed left kidney	colchicine + LMWH, discontinuation of warfarin, urgent left sided nephrectomy due to severe bleeding	general improvement, resolution of bleeding, no data on kidney function
**Mendonca S et al., 2017. [19]**	33	m	warfarin	PE	/	5.3/-	79	230	hematuria	twenty-two days	none	YES: glomerular congestion with RBCs, diffuse tubular damage with large and occlusive RBC casts, interstitial hemosiderin laden macrophages, vascular hyalinosis	discontinuation of warfarin	improvement of kidney function
**Nagasako Y et al., 2017. [20]**	83	m	warfarin	atrial fibrillation	/	1.44/-	85.7	212	gross hematuria	twelve years	invasive urothelial carcinoma of the right renal pelvis - previously unknown, previous intermittent gross hematuria, removed bladder urothelial carcinoma	YES, after nephrectomy: tubular obstruction by red blood cell casts, acute tubular injury of the non-tumor tissue	discontinuation of warfarin therapy, right nephro-ureterectomy due to papillary right renal tumor	improvement of kidney function

**Rawala MS et al., 2019. [21]**	61	m	warfarin	atrial fibrillation mech. mitral valve	/	3.5/-	179	601	gross hematuria	U	none	YES: mild IgAN, diffuse tubular injury, RBC casts inside the tubules	U	U

Abbreviations: AC, anticoagulant; IgAN; IgA nephropathy; TIN, tubulointerstitial nephritis; GN glomerulonephritis; U, unknown; GI, gastrointestinal; INR, international normalized ratio; aPTT, activated partial thromboplastin time; SCr, serum creatinine; IgA, immunoglobulin A; RBC, red blood cell; CKD, chronic kidney disease; ANCA, anti-neutrophil cytoplasmic antibody; CrCl, creatinine clearance; ATN, acute tubular necrosis; NH-MCL, non-Hodgkin- mantle cell lymphoma; IF, immunofluorescence; AKI, acute kidney injury; LMWH, low-molecular-weight-heparin, DVT, deep venous thrombosis; pres., presumed, PE, pulmonary embolism; mech., mechanical, postreptoc., postreptococcal.

Supplementary Table 3: International case series with kidney biopsy findings from 2009 to 2019 (source Pubmed).

Reference	*N*	Anticoagulation	Age (years), mean	Male (%)	Prevalence of CKD (%)	Prevalence of diabetes mellitus (%)	Prevalence of hypertension (%)	Positive ANCA/SLE (%)	SCr baseline (µmol/l), mean	SCr at diagnosis (µmol/l), mean	Most common kidney biopsy findings	Outcome
**Brodsky SV et al., 2009. [22]**	9	warfarin	61.6	55.5	100	NA	NA	NA	106	380	RBCs within the tubular lumina with formation of occlusive RBC casts in all patients, underlying kidney disease in all patients	no recovery of kidney function in five patients, four patients remained on dialysis
**Brodsky SV et al., 2019. [23]**	30	warfarin/DOAC	61.2	60	NA	13	23	26	114	367.8	various pre-existent pathological changes in the glomeruli, features of ATN and RBC casts in the tubules in all biopsies	NA

Abbreviations: CKD, chronic kidney disease; ANCA, anti-neutrophil cytoplasmic antibody; SLE, systemic lupus nephritis; SCr, serum creatinine; RBC, red blood cell; ATN, acute tubular necrosis.

Reference list

1. Góis, M.; Azevedo, A.; Carvalho, F.; Nolasco, F. Anticoagulant-related nephropathy in a patient with IgA nephropathy. *BMJ case reports* **2017**, *2017*, doi:10.1136/bcr-2016-218748.
2. Golla, A.; Goli, R.; Nagalla, V.K.; Kiran, B.V.; Raju, D.S.B.; Uppin, M.S. Warfarin-related Nephropathy. *Indian J Nephrol* **2018**, *28*, 378-381, doi:10.4103/ijn.IJN_3_17.
3. Brodsky, S.V.; Mhaskar, N.S.; Thiruveedi, S.; Dhingra, R.; Reuben, S.C.; Calomeni, E.; Ivanov, I.; Satoskar, A.; Hemminger, J.; Nadasdy, G., et al. Acute kidney injury aggravated by treatment initiation with apixaban: Another twist of anticoagulant-related nephropathy. *Kidney research and clinical practice* **2017**, *36*, 387-392, doi:10.23876/j.krcp.2017.36.4.387.
4. Escoli, R.; Santos, P.; Andrade, S.; Carvalho, F. Dabigatran-Related Nephropathy in a Patient with Undiagnosed IgA Nephropathy. *Case reports in nephrology* **2015**, *2015*, 298261, doi:10.1155/2015/298261.
5. Kalaitzidis, R.G.; Duni, A.; Liapis, G.; Balafa, O.; Xiromeriti, S.; Rapsomanikis, P.K.; Elisaf, M.S. Anticoagulant-related nephropathy: a case report and review of the literature of an increasingly recognized entity. *International urology and nephrology* **2017**, *49*, 1401-1407, doi:10.1007/s11255-017-1527-9.
6. Li, X.; Cheung, C.Y. Dabigatran causing severe acute kidney injury in a patient with liver cirrhosis. *CEN Case Rep* **2019**, *8*, 125-127, doi:10.1007/s13730-019-00378-4.
7. Ikeda, M.; Tanaka, M.; Shimoda, S.; Saita, H.; Nishikawa, S.; Shimada, H.; Taniguchi, K.; Hagihara, K.; Iwanari, S.; Takeoka, H. Dabigatran-induced anticoagulant-related nephropathy with undiagnosed IgA nephropathy in a patient with normal baseline renal function. *CEN Case Rep* **2019**, *8*, 292-296, doi:10.1007/s13730-019-00410-7.
8. Sharfuddin, N.; Nourbakhsh, M.; Box, A.; Benediktsson, H.; Muruve, D.A. Anticoagulant Related Nephropathy Induced by Dabigatran. *Case reports in nephrology* **2018**, *2018*, 7381505, doi:10.1155/2018/7381505.
9. Krátká, K.; Havrda, M.; Honsová, E.; Rychlík, I. Bioptically Proven "Anticoagulation-Related Nephropathy" Induced by Dual Antiplatelet Therapy. *Case Rep Nephrol Dial* **2018**, *8*, 216-222, doi:10.1159/000493093.
10. Fujino, Y.; Takahashi, C.; Mitsumoto, K.; Uzu, T. Rivaroxaban-related acute kidney injury in a patient with IgA vasculitis. *BMJ case reports* **2019**, *12*, doi:10.1136/bcr-2018-227756.
11. Oliveira, M.; Lima, C.; Góis, M.; Viana, H.; Carvalho, F.; Lemos, S. Rivaroxaban-related nephropathy. *Portuguese Journal of Nephrology & Hypertension* **2017**, *31*, 212-216.
12. Ng, C.Y.; Tan, C.S.; Chin, C.T.; Lim, S.L.; Zhu, L.; Woo, K.T.; Tan, P.H. Warfarin related nephropathy: a case report and review of the literature. *BMC Nephrol* **2016**, *17*, 15, doi:10.1186/s12882-016-0228-4.
13. Bento, C.P.; Soares, M.; Molin, C.; Martins, L.; Martins, J.; Mazza, M.; Riella, M. [Case report: end stage renal disease in a chronic kidney patient on warfarin therapy]. *J Bras Nefrol* **2015**, *37*, 275-278, doi:10.5935/0101-2800.20150043.
14. Behera, S.K.; Xavier, A.S.; Selvarajan, S.; Munuswamy, H.; Haridasan, S.; Srinivas, B.H. Acenocoumarol as an alternative anticoagulant in a patient with warfarin-related nephropathy. *Br J Clin Pharmacol* **2018**, *84*, 1068-1071, doi:10.1111/bcp.13541.
15. Santos, C.; Gomes, A.M.; Ventura, A.; Almeida, C.; Seabra, J. An unusual cause of glomerular hematuria and acute kidney injury in a chronic kidney disease patient during warfarin therapy. *Nefrologia* **2013**, *33*, 400-403, doi:10.3265/Nefrologia.pre2012.Oct.11617.

16. Zerah, L.; Brochériou, I.; Galichon, P.; Peltier, J.; Hertig, A. [Warfarin-related nephropathy: a case report]. *Rev Med Interne* **2015**, *36*, 51-54, doi:10.1016/j.revmed.2013.09.006.
17. Larpparisuth, N.; Cheunsuchon, B.; Chawanasuntorapoj, R.; Vasuvattakul, S.; Vareesangthip, K. Warfarin related nephropathy: the first case report in Thailand. *J Med Assoc Thai* **2015**, *98*, 212-216.
18. Remková, A.; Milatová, E.; Fuchsbergerová, M. A severe renal bleeding as a complication of coumarin therapy in Behçet's disease. *Blood Coagul Fibrinolysis* **2010**, *21*, 487-490, doi:10.1097/MBC.0b013e328338cded.
19. Mendonca, S.; Gupta, D.; Valsan, A.; Tewari, R. Warfarin related acute kidney injury: A case report. *Indian J Nephrol* **2017**, *27*, 78-80, doi:10.4103/0971-4065.177142.
20. Nagasako, Y.; Fujii, A.; Furuse, S.; Saito, K.; Mise, N. Warfarin-related nephropathy in a patient with renal pelvic cancer. *Clin Nephrol Case Stud* **2017**, *5*, 5-8, doi:10.5414/cncs108862.
21. Rawala, M.S.; Ahmed, A.S.; Khan, M.Y.; Riaz, M.N.; Eltoukhy, A. Supratherapeutic International Normalized Ratio causing Nephropathy: A Rare Adverse Effect of Warfarin. *Cureus* **2019**, *11*, e5201, doi:10.7759/cureus.5201.
22. Brodsky, S.V.; Satoskar, A.; Chen, J.; Nadasdy, G.; Eagen, J.W.; Hamirani, M.; Hebert, L.; Calomeni, E.; Nadasdy, T. Acute kidney injury during warfarin therapy associated with obstructive tubular red blood cell casts: a report of 9 cases. *American journal of kidney diseases : the official journal of the National Kidney Foundation* **2009**, *54*, 1121-1126, doi:10.1053/j.ajkd.2009.04.024.
23. Brodsky, S.V.; Satoskar, A.; Hemminger, J.; Rovin, B.; Hebert, L.; Ryan, M.S.; Nadasdy, T. Anticoagulant-Related Nephropathy in Kidney Biopsy: A Single-Center Report of 41 Cases. *Kidney Med* **2019**, *1*, 51-56, doi:10.1016/j.xkme.2019.03.002.
